# Phytotoxicity and allelopathic potential of *Juglans regia* L. leaf extract

**DOI:** 10.3389/fpls.2022.986740

**Published:** 2022-10-05

**Authors:** Tijana Đorđević, Rada Đurović-Pejčev, Marija Stevanović, Marija Sarić-Krsmanović, Ljiljana Radivojević, Ljiljana Šantrić, Jelena Gajić-Umiljendić

**Affiliations:** ^1^Laboratory of Chemistry and Ecotoxicology, Institute of Pesticides and Environmental Protection, Belgrade, Serbia; ^2^Laboratory of Weed Research, Institute of Pesticides and Environmental Protection, Belgrade, Serbia

**Keywords:** walnut leaf extract (WLE), phenols, allelopathy, germination, seedling growth, oxidative stress

## Abstract

Implementation of plant extracts that are rich in phytochemicals and have the allelopathic potential for weed management could help reduce the use of chemical herbicides. The present study investigated the herbicidal potential of walnut (*Juglans regia* L.) leaf extract (WLE) against two weeds, *Amaranthus retroflexus* L. and *Chenopodium album* L., by testing *in vitro* their seed germination and seedling growth, and then evaluated *in vivo* the oxidative stress of potted plants. The effects of the walnut leaf extract were also tested on maize (*Zea mays* L.) to eliminate possible negative impacts on a cultivated plant. Total phenolic acids and total flavonoid content in the extract were determined in prior bioassays, followed by separation and analysis of flavonoids and phenolic acids by high-performance liquid chromatography (HPLC). Phytochemical analysis revealed that the obtained extract was notably rich in phenolic compounds, while HPLC analysis confirmed the presence of (+)-catechin, luteolin, myricetin, rutin, (–)-epicatechin, genistin, protocatechuic acid, and caffeic acid as major extract components. The results obtained in bioassays revealed a significant negative impact of the walnut leaf extract on germination and seedling growth of the tested weeds, as well as significant oxidative stress in weeds grown in pots. Although it affected the maize seedling growth *in vitro* similar to the tested weeds, maize germination was less sensitive to treatment, and the extract did not have a significant negative impact in terms of oxidative stress in maize plants grown in pots. The findings show that walnut leaf extract may have a promising role in replacing chemical herbicides in maize.

## Introduction

The yield of major crops, as well as the quality of harvested products, are severely compromised by weed infestation. Considering maize, one of the global staple crops, interference from weeds is a critical factor that limits its field productivity. Weeds primarily impact maize yield through competition for limited natural resources (sunlight, water, and nutrients), but they also compromise crop production by interfering with harvest efficiency and serving as alternative hosts for various pests.

Conventional weed management strategies, which imply extensive use of chemical herbicides, restrict weed growth for a short period but weeds mostly reappear in the field soon after. Besides, along with their negative impact on human and animal health and the environment through the increment of toxic herbicide residues in products, soil, and groundwater, herbicide resistance developed by important weed species is becoming a major issue for chemical control strategy. The latest updates show 512 cases of herbicide-resistant weeds globally; while 266 species have developed resistance to 165 herbicides with 21 of the 31 known herbicide sites of action, herbicide-resistant weeds have been reported in 96 crops in 71 countries (Heap, 2022). Considering these negative impacts of conventional weed control methods and increasingly frequent incidents of herbicide resistance, the tendency toward finding potential allelochemicals as effective and safer alternatives to synthetic herbicides is rapidly increasing.

Against this background, non-chemical weed control strategies, particularly weed management methods based on allelopathy have turned out to be one of the most effective (Jabran and Farooq, 2013). Allelopathy as a phenomenon relies on allelochemicals produced by plants. Allelochemicals are secondary metabolites such as phenolic compounds, sterols, terpenes, alkaloids, saponins, essential oils, fatty acids, or others, which are not essential for plant growth and development but are required for the plant's interaction with the environment. Under natural environmental conditions, allelopathy occurs when chemicals produced by plants are released onto neighboring plants in the same population, affecting their germination, development, reproduction, and survival. Various allelopathic compounds, extracted from numerous different plant species, have already been tested for potential use in weed control within organic agricultural systems, and many of them have demonstrated destructive effects on some weed plants, inhibiting their seed germination and shoot and root elongation and disrupting their photosynthesis and water/nutrient uptake (Grisi et al., 2015; Jang et al., 2018; Puig et al., 2021). As the half-lives of natural compounds with allelopathic capacities are usually short-lived, there would be no concerns regarding residues in soil. This advantage, along with their efficiency, is one of the reasons why numerous studies report that secondary metabolites of certain plants could become a significant component in weed control strategies. Implementation of plant extracts rich in phytochemicals that have allelopathic potential in weed management could reduce the usage of chemical herbicides, thus promoting the protection of human health and preservation of the environment.

Phenolic compounds are one of the most widely occurring groups of phytochemicals, which have considerable physiological and morphological importance in plants and exhibit a wide range of physiological properties. Phenolic compounds act as antioxidant, anti-inflammatory, and anti-microbial agents that protect the organism producing them. Besides, phenols synthesized by plants also tend to inhibit the growth of other plant competitors, thereby involving phytochemicals in plant allelopathy (Heleno et al., 2015). Not only can phenolic compounds interfere with the activities of respiratory enzymes involved in seed germination (Muscolo et al., 2001) or modify the activity of the growth hormone gibberellic acid, thereby inhibiting germination (Olofsdotter, 2001), but phytochemicals can also influence the expression of specific genes associated with root tissue differentiation and inhibit root development (Franco et al., 2015). Additionally, phenols can be involved in reactive oxygen species (ROS) production and suppression of antioxidant enzyme activity (Inzé and Montagu, 2001; Gniazdowska and Bogatek, 2005), as well as provoke enhanced ROS production in competitor plants, which exceeds the capacity of attenuated antioxidant defense enzymes, resulting in oxidative stress and plant cell death (Chaki et al., 2020). All those abilities are reasons for plant phenolic compounds to be considered a major source of allelochemicals (ŠeŽiene et al., 2017).

In the context of recent research, the present study was designed to investigate the herbicidal potential of walnut (*Juglans regia* L.) leaf extract against two major weeds, *Amaranthus retroflexus* L. (redroot pigweed) and *Chenopodium album* L. (lamb's quarters), by evaluating their seed germination and seedling growth *in vitro*, and oxidative stress in growing weed plants *in vivo*. As those weeds are common in maize crops, the effect of walnut leaf extract was also tested *in vitro* and *in vivo* on maize (*Zea mays* L.) plants to eliminate possible negative impacts on cultivated plants. Before the bioassays, phytochemical analysis of the obtained walnut leaf extract was performed, focusing on total phenolic acid content analysis (TPC), total flavonoid content analysis (TFC), and separation and analysis of flavonoids and phenolic acids by HPLC, to identify and characterize potent phenolic allelochemicals in the extract.

## Materials and methods

### Plant material and plant extraction

Fresh walnut leaves were collected from mature trees grown in Vojvodina Province, Serbia, in April 2019, and then shade dried, stored in paper bags to protect them from light, and milled just before phenolic extraction.

A mix of methanol/acetone/water (40/40/20 v/v/v) was used as an extraction solvent. The powdery material was soaked in the solvent in a 1:5 solid-to-volume ratio and sonicated for 15 min in an ultrasonic bath. Extraction was performed in triplicate and aliquots from the three extractions were merged after centrifugation (3,000 rpm, 10 min) and filtration (Whatman filter paper grade 1). The extract was evaporated to dryness at 50°C using a vacuum rotary evaporator. Residues were dissolved in distilled water, freeze-dried, and kept at −20°C for future analysis.

### Phytochemical analyses of extract

#### Total phenolic content

The total phenolic content of walnut leaf extract was determined by a modified Folin-Ciocalteu method (Singleton and Rossi, 1956). Dry walnut leaf extract (0.25 g) was dissolved in distilled water (25 ml). A volume of 100 μl of extract solution was mixed with 0.5 ml of Folin-Ciocalteu reagent and 6 ml of distilled water in test tubes and vortexed well. A volume of 2 ml of 15% Na_2_CO_3_ was then added to the solution, the volume was adjusted up to 10 ml with distilled water, and the solution was incubated for 60 min at room temperature. The absorbance was measured at 760 nm wavelength by spectrophotometer (Perkin Elmer, Lambda Bio). A solution containing only the reagents except the walnut leaf extract was considered a blank. Gallic acid (GAE) was used as an equivalent for calibration curve preparation, and the results were expressed as mg gallic acid per g of dry extract (mg GAE g^−1^ d.e.).

#### Total flavonoid content

The total flavonoid content of walnut leaf extract was determined using a modified aluminum chloride colorimetric assay (Woisky and Salatino, 1998). Dry walnut leaf extract (0.125 g) was dissolved in methanol (25 ml). A volume of 375 μl of extract solution was mixed with 1.5 ml of 5% NaNO_2_ solution in test tubes and incubated at room temperature for 6 min. A volume of 150 μl of 10% AlCl_3_ solution was added, vortexed well, and left for another 5 min. A volume of 750 μl of 1M NaOH solution was added and further incubated for 15 min. The absorbance was measured at 510 nm wavelength by spectrophotometer. A solution containing only the reagents except the walnut leaf extract was considered a blank. Quercetin (QE) was used as an equivalent for calibration curve preparation, and results were expressed as mg quercetin per g of dry extract (mg QE g^−1^ d.e.).

#### Separation and analysis of flavonoids and phenolic acids

Dry walnut leaf extract (0.5 g) was dissolved in methanol to obtain the final sample concentration of ~10 mg ml^−1^. Phenolic compound analyses were performed on an HPLC system Shimadzu Prominence equipped with a photodiode array (PDA) detector. Chromatographic separation was achieved by the Zorbax Eclipse XDB C18 column (3.6 μm particle size, 4.6 × 150 mm) maintained at 40°C. The mobile phases were 1% acetic acid in distilled water (solvent A), methanol (solvent B), and acetonitrile (solvent C) in gradient (0 min – 90% A, 10% B, 0% C; 10 min – 80% A, 16% B, 4% C; 25 min – 75% A, 20% B, 5% C; 30 min – 65% A, 30% B, 5% C; 31 min – 40% A, 60% B, 0% C; 37 min – 35% A, 45% B, 20% C; 50 min – 20% A, 0% B, 80% C; 55–57 min – 0% A, 0% B, 100% C; 58–65 min – 90% A, 10% B, 0% C), with the flow rate of 0.8 ml min^−1^. Detection was performed by scanning from 200 to 400 nm and a volume of 20 μl of standards and samples was injected. All phenolic acids standards (gallic acid, 4-hydroxybenzoic acid, protocatechuic acid, vanillic acid, cinnamic acid, ferulic acid, caffeic acid, p-coumaric acid) and flavonoids standards [(+)-catechin, (–)-epicatechin, (–)-epigallocatechin, quercetin, myricetin, rutin, luteolin, daidzin, genistin, and glycitin], used for external quantification of compounds, were purchased from Sigma Aldrich and dissolved in methanol. Stock solutions of individual compounds were prepared at a concentration of 1.0 mg ml^−1^ in methanol. The stock mixture standard (working solution containing all 18 phenol compounds) was obtained by mixing and diluting the stock standards. Retention times and spectral data of standards were noted for reference. The composite mixtures of all phenolic compounds at appropriate concentrations were used to spike samples for qualification confirmation. Phenolic compounds quantification was achieved by recording the absorbance in chromatograms relative to external standards, with detection at 260, 270, 280, 320, and 360 nm. Concentrations of compounds were expressed as mg g^−1^ of dry extract.

### *In vitro* evaluation of seed germination and seedling growth

A petri-dish experiment was set up for *A. retroflexus, C. album*, and maize under controlled conditions. Seeds of *A. retroflexus* were collected in fields around Jakovo (Belgrade, Central Serbia) in October 2020. Seeds of *C. album* were collected in fields around Padinska Skela (Belgrade, Central Serbia) in October 2020. The seeds were cleaned and stored in paper bags in the laboratory at a temperature of 20–22°C. Maize (hybrid NS3022) seeds were provided by the Institute of Field and Vegetable Crops, Novi Sad, Serbia. Before the experiment, the seeds were surface sterilized for 3 min in a 5% aqueous solution of sodium hypochlorite and washed several times with distilled water. Twenty-five disinfected seeds were placed into each Petri dish lined with sterilized filter paper disks. Walnut leaf extract was diluted in distilled water at 0.5, 0.75, and 1% (w/v) concentrations, 5 ml was applied to each Petri dish with weed seeds, and 10 ml was applied to each Petri dish with maize seeds. Distilled water was applied to control plates. All dishes were sealed with parafilm to avoid evaporation. The dishes were placed in an incubator (Binder CE) at 27 ± 1°C and kept in darkness. After a period of 7 days, germination percentage was calculated, and early seedling growth was measured as radial and shoot length. The experiment design was a randomized complete block with four replications, repeated twice and data were combined for analysis.

### *In vivo* evaluation of oxidative stress in growing plants

Oxidative stress in plants was evaluated in a pot experiment. The sowing substrate consisted of garden humus and sterilized sand at a 2:1 ratio. A 250 g sample of sowing substrate was measured and poured into each pot. The weed and crop seeds were surface sterilized for 3 min in a 5% aqueous solution of sodium hypochlorite and washed several times with distilled water. The seeds were individually sown in pots (ø 12 cm) and the pots were then put in a growing chamber under controlled conditions (photoperiod 14 h/10 h, temperature 26°C/day, 21°C/night, humidity 60–70%, and light intensity 300 μE/m^2^s). Sterilized water was used for daily irrigation. Three plants remained in each pot after thinning. Walnut leaf extract was diluted with sterile distilled water to provide 0.75 and 1% (w/v) concentrations, and 3 ml of each concentration were pipetted and transferred to a thin-layer chromatography sprayer which was connected to a compressor. Plants at the growth stage of two-three true leaves (12–13 BBCH scale) were treated uniformly under 1–2 bars of pressure. Control plants were sprayed with sterile distilled water. The evaluation of oxidative stress was conducted 7 and 14 days after treatment (DAT). The experiment design was a randomized complete block with four replications, repeated twice and data were combined for analysis.

### Determination of oxidative stress parameters

To determine the oxidative stress parameters, i.e., the intensity of lipid peroxidation and changes in the activity of antioxidant enzymes, all treated plants (three from each pot) were harvested carefully one by one at each sampling date to prepare plant extracts. Each sample, consisting of 1 g of fresh plant material (shoots), was transferred to a mortar (previously cooled in ice) and after adding ~2% w/w of polyvinylpyrrolidone (PVP) was crushed and homogenized with 5 ml of cooled extraction buffer [0.1 M potassium phosphate buffer (pH 7.0) amended with 1 mM ethylenediaminetetraacetic acid (EDTA) and 5 mM sodium ascorbate]. The homogenized material was centrifuged at 18,000×*g* for 15 min at 4°C and supernatant was used as an enzymatic source. All analyses were performed in triplicate.

#### Lipid peroxidation

The intensity of lipid peroxidation (LP) was determined by estimating malondialdehyde (MDA), a decomposition product of the peroxidized polyunsaturated fatty acid composition of membrane lipid, using thiobarbituric acid (TBA) as the reactive material. This method is based on measuring the increase in absorbance due to the formation of the malondialdehyde-thiobarbituric acid (TBA-MDA) complex (Heath and Packer, 1968). Plant extract (250 μl) was incubated with a reaction medium: 1 ml of 20% trichloroacetic acid (TCA, w/v) containing 1% TBA (w/v) for 30 min at 95°C. The reaction was stopped by cooling on ice for 10 min, and the product was centrifuged at 18,000×*g* for 15 min. The absorbance of the supernatant was measured at 532 nm and was corrected for non-specific absorbance at 600 nm and absorbance of blank (reaction medium) at those wavelengths. The amount of malondialdehyde was calculated using the molar extinction coefficient of 155 mM^−1^ cm^−1^. The total amount of products of lipid peroxidation (TBA-reactive substances—TBARS) was expressed as μmol MDA per gram of fresh weight (μmol MDA g^−1^ f.w.).

#### Superoxide dismutase activity

Superoxide dismutase (SOD) (EC 1.15.1.1) activity was determined as an ability of plant enzyme extracts to inhibit the photochemical reduction of nitrotetrazolium blue (NBT) (Beyer and Fridovich, 1987). The reaction medium was prepared by mixing 890 μl of the reaction mixture [50 mM potassium phosphate buffer (pH 7.8) amended with 0.1 mM EDTA, 13 mM methionine, and 75 μM NBT], 10 μl of 1 mM riboflavin, and 100 μl of plant extract, pure or diluted with extraction buffer. The reaction medium was kept under a fluorescent lamp (30 W) for 10 min and absorbance was read at 560 nm. The reaction medium prepared with 100 μL of extraction buffer instead of enzyme extract was used as control. One unit of SOD activity (U) was defined as the amount of enzymes required to inhibit NBT reduction by 50%. Enzyme activity was expressed as U per gram of fresh weight (U g^−1^ f.w.).

#### Guaiacol peroxidase activity

Guaiacol peroxidase (GPOD) (EC 1.11.1.7) activity was measured using guaiacol as a substrate. This method is based on monitoring absorbance change due to guaiacol oxidation, i.e., tetraguaiacol formation (Siegel and Galston, 1967). The reaction medium was prepared by mixing 900 μl of the reaction mixture [0.2 M potassium phosphate buffer (pH 5.8) amended with 5 mM guaiacol and 5 mM hydrogen peroxide] and 100 μl of plant enzyme extract. Change in the absorbance at 470 nm was monitored for 2 min (recording absorbance at 15 s intervals). Enzyme activity was calculated using the molar extinction coefficient of 2.47 mM^−1^ cm^−1^. One unit of GPOD activity (U) was defined as μmol of tetraguaiacol formed per minute. Enzyme activity was expressed as U per gram of fresh weight (U g^−1^ f.w.).

#### Ascorbate peroxidase activity

Ascorbate peroxidase (APX) (EC 1.11.1.1) activity was measured using ascorbic acid as a substrate. This method is based on monitoring absorbance changes due to ascorbate oxidation, i.e., monodehydroascorbic acid (MDHA) formation (Nakano and Asada, 1981). The reaction medium was prepared by mixing 950 μl of the reaction mixture [50 mM potassium phosphate buffer (pH 7.0) amended with 0.1 mM EDTA, 0.1 mM hydrogen peroxide, and 0.5 mM ascorbic acid] and 50 μl of plant enzyme extract. Absorbance change at 290 nm was monitored for 2 min (recording absorbance at intervals of 15 s). Enzyme activity was calculated using the molar extinction coefficient of 2.8 mM^−1^ cm^−1^. One unit of APX activity (U) was defined as μmol of MDHA formed per minute. The activity of the enzyme was expressed as U per gram of fresh weight (U g^−1^ f.w.).

#### Catalase activity

Catalase (CAT) (EC 1.11.1.6) activity was measured by a method based on monitoring absorbance changes due to hydrogen peroxide dissociation (Aebi, 1974). The reaction medium was prepared by mixing 950 μl of the reaction mixture [50 mM potassium phosphate buffer (pH 7.0) amended with 20 mM hydrogen peroxide] and 50 μl of plant enzyme extract. Change in the absorbance at 240 nm was monitored for 2 min (recording absorbance at 15 s intervals). The enzyme activity was calculated using the molar extinction coefficient of 0.036 mM^−1^ cm^−1^. One unit of CAT activity (U) was defined as the μmol of dissociated hydrogen peroxide per minute. Enzyme activity was expressed as U per gram of fresh weight (U g^−1^ f.w.).

### Statistical analysis

All chemical and biochemical measurements were performed in triplicates and results were expressed as mean ± standard deviation. Data from biochemical measurements were analyzed by one-way analysis of variance (ANOVA) using GraphPad Prism. When *F*-values were statistically significant (*p* < 0.05) treatments were compared using Tukey's Honestly Significant Difference (HDS) test. The designs of the germination bioassay and pot experiment were randomized complete blocks with four replications, repeated twice, and data were combined for analysis. Data from the germination bioassay were analyzed by one-way analysis of variance (ANOVA) using STATISTICA 8.0. software package. When F-values were statistically significant (*p* < 0.05) treatments were compared by Fisher's Least Significant Difference (LSD) test.

## Results

### Phytochemical analyses of walnut leaf extract

The results obtained from the analysis of total phenolic and total flavonoid contents showed the presence of their significant amounts in the obtained walnut leaf extract. Total phenolic compound content was 113.0 ± 4.3 mg GAE g^−1^ dry extract, while total flavonoid compound content was 634.3 ± 6.3 mg QE g^−1^ dry extract. As a confirmation of TPC and TFC results, an HPLC analysis of WLE revealed that flavonoids were overall more abundant than phenolic acids (Table 1).

**Table 1:** Quantification of phenols in obtained walnut leaf extract.

**Content of phenolic compounds**		
**Chemical class**	**Compound**	**Concentration**
		**(mg/g of dry extract)**
Flavones	Luteolin	12.93 ± 0.09
Flavan-3-ols	(+)-Catechin	36.78 ± 0.12
	(–)-Epicatechin	7.11 ± 0.09
	(–)-Epigallocatechin	2.18 ± 0.01
Flavonols	Quercetin	4.08 ± 0.04
	Myricetin	10.98 ± 0.08
	Rutin	8.01 ± 0.07
Isoflavones	Daidzin	0.44 ± 0.02
	Genistin	7.44 ± 0.08
	Glycitin	0.30 ± 0.01
Hydroxybenzoic acids	Vanillic acid	1.44 ± 0.02
	4-Hydroxybenzoic acid	0.77 ± 0.02
	Gallic acid	1.40 ± 0.02
	Protocatechuic acid	7.86 ± 0.07
Hydroxycinnamic acids	Caffeic acid	2.91 ± 0.05
	Cinnamic acid	0.96 ± 0.04
	Ferulic acid	0.72 ± 0.03
	p-Coumaric acid	0.22 ± 0.02

Values are presented as mean ± SD (*n* = 3).

Of the 18 detected phenolic compounds, the largest contribution to high amounts of flavonoids in the extract was mainly from (+)-catechin, then by luteolin and myricetin, as well as rutin, (–)-epicatechin, and genistin, while other flavonoid compounds were detected at moderate or lower rates. Regarding phenolic acids, hydroxybenzoic acids were present at a higher rate in the extract, protocatechuic acid being the most abundant, while hydroxycinnamic acids were represented at a moderate to low rate with caffeic acid at the highest level among them.

### *In vitro* evaluation of WLE effect on seed germination and seedling growth

**Figure 1:**
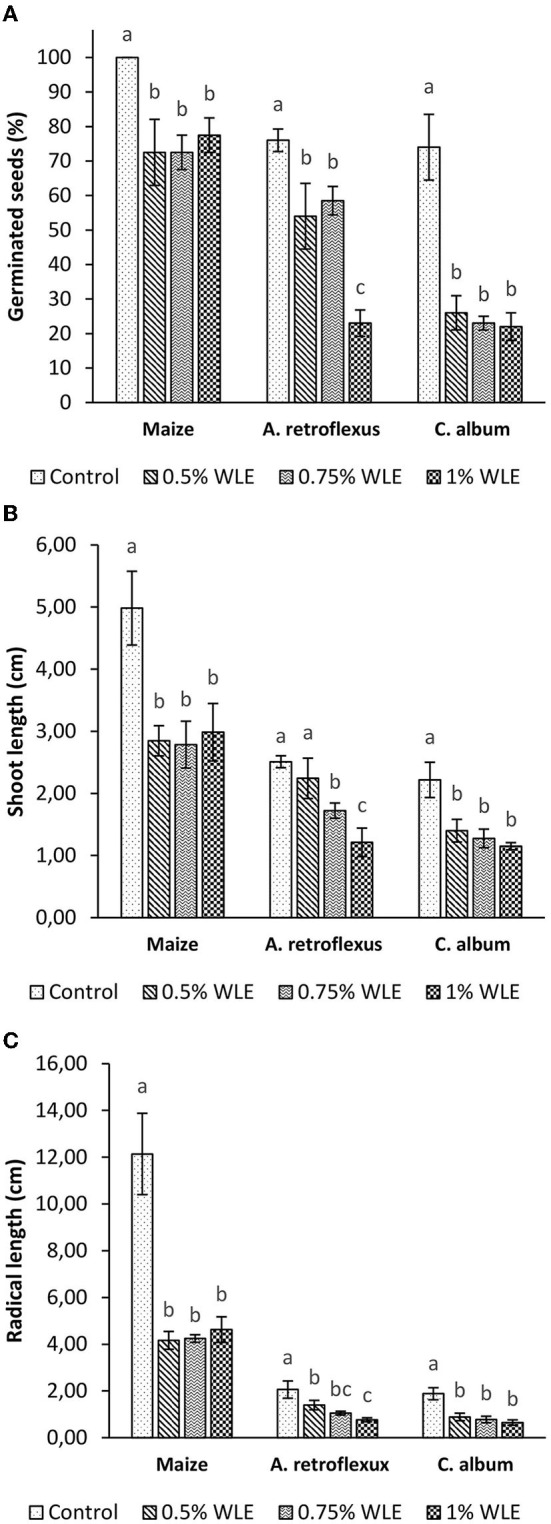
Seed germination **(A)**, shoot growth **(B)**, and radical growth **(C)** of maize and weeds - *Amaranthus retroflexus* and *Chenopodium album* as a response to treatment with 0.5, 0.75, and 1% walnut leaf extract. Data are presented as mean ± SD. Values marked with different letters within a group differ significantly based on Fisher's LSD test (*p* ≤ 0.05).

The effects of different concentrations of walnut leaf extract on seed germination and seedling growth of the weeds *A. retroflexus* and *C. album*, as well as maize, are presented in Figure 1.

The results clearly show that nearly 80% of *A. retroflexus* control seeds germinated. The response to all walnut leaf extract concentrations was significant. Seed germination was moderately inhibited by treatments with 0.5 and 0.75% WLE (29 and 22% respectively, without statistically significant differences between the two results), while high germination inhibition (70%) was found for treatment with the highest concentration (1% WLE). Regarding seedling growth, walnut leaf extract had a significant effect on shoot growth of *A. retroflexus* only when higher concentrations (0.75 and 1%) were applied, so that shoot length was reduced by 31 and 52%, respectively. Radical growth was more affected by WLE treatment, and it was 32, 49, and 63% for this weed, respectively for growing concentrations. Regarding *C. album*, it responded to treatment with walnut leaf extract almost in the same manner as the former weed. Germination of control seeds was ~75%, while WLE inhibited germination up to 70%, and the result was similar for the three concentrations (65, 69, and 70%, respectively for growing concentrations). Shoot elongation was moderately affected by WLE and without statistically significant differences between the three concentrations, so shoot growth inhibition was 34, 40, and 46%, respectively. Walnut leaf extract had a stronger effect on *C. album* radical growth than on shoot elongation. The decrease in radical length was 52, 58, and 64%, respectively for the three concentrations of WLE, again without statistically significant differences. Maize had the highest germination rate of control seeds (100%), and its germination was significantly less affected by walnut leaf extract. Inhibition of germination after treatment with WLE was approximately 30% for all three concentrations. Considering seedling growth, however, the effect was similar to the impact on the tested weeds. Walnut leaf extract had a slighter effect on shoot growth (~45% inhibition) compared to radical growth (~65% of inhibition). No differences between treatments with the three concentrations of WLE were noted.

### *In vivo* evaluation of WLE effect on oxidative stress in growing plants

**Figure 2:**
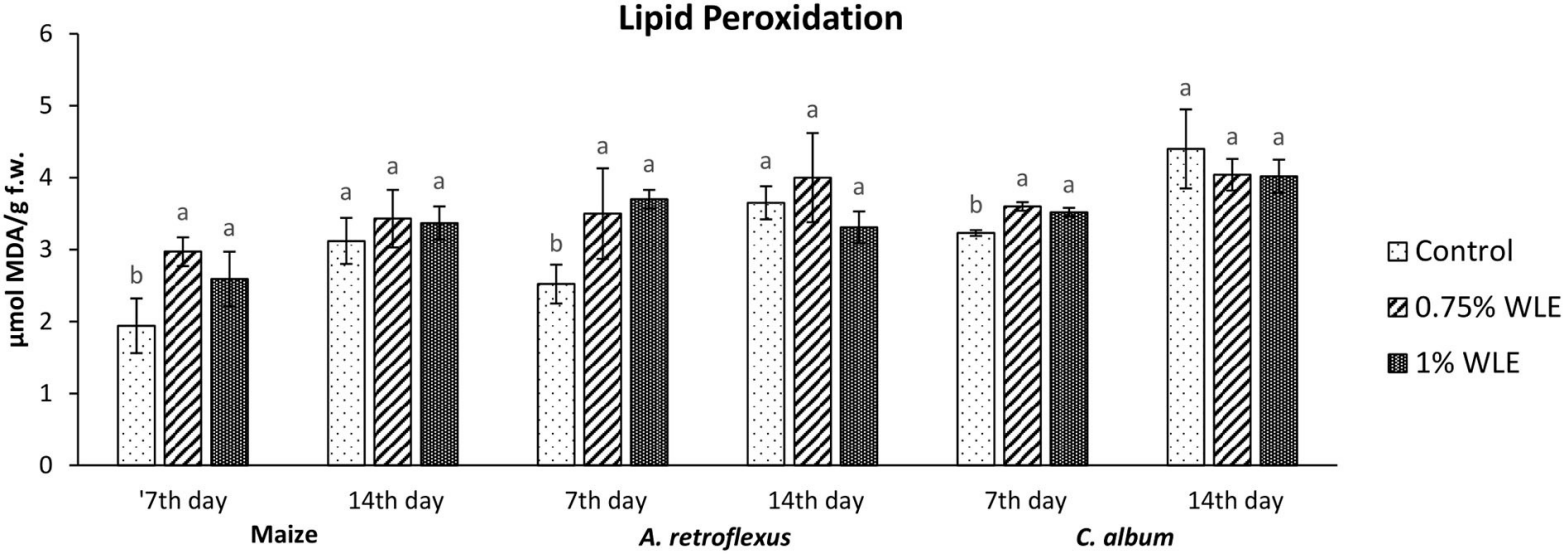
Lipid peroxidation in maize and weeds - *Amaranthus retroflexus* and *Chenopodium album* as a response to treatment with 0.75 and 1% walnut leaf extract, on 7^th^ and 14^th^ day after treatment. Data are presented as mean ± SD. Values marked with different letters within a group differ significantly based on Tukey's HSD test (*p* ≤ 0.05).

**Figure 3:**
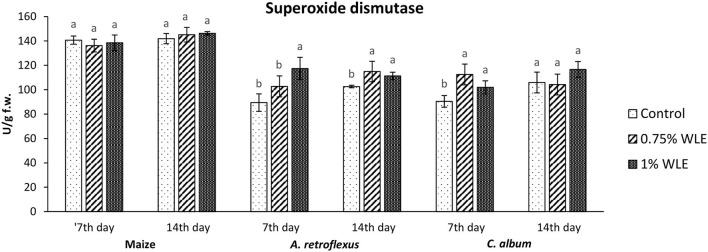
Activity of antioxidant enzyme superoxide dismutase (SOD) in maize and weeds - *Amaranthus retroflexus* and *Chenopodium album* as a response to treatment with 0.75 and 1% walnut leaf extract, on the 7^th^ and 14^th^ day after treatment. Data are presented as mean ± SD. Values marked with different letters within a group differ significantly based on Tukey's HSD test (*p* ≤ 0.05).

**Figure 4:**
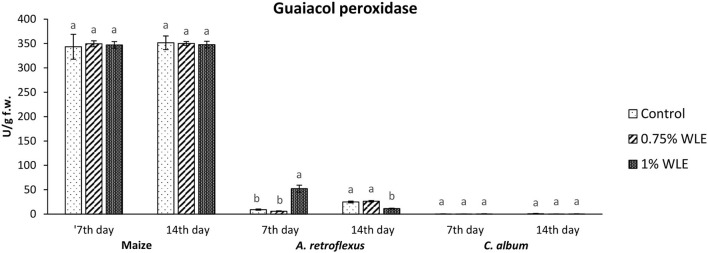
Activity of antioxidant enzyme guaiacol peroxidase (GPOD) in maize and weeds - *Amaranthus retroflexus* and *Chenopodium album* as a response to treatment with 0.75 and 1% walnut leaf extract, on the 7^th^ and 14^th^ day after treatment. Data are presented as mean ± SD. Values marked with different letters within a group differ significantly based on Tukey's HSD test (*p* ≤ 0.05).

**Figure 5:**
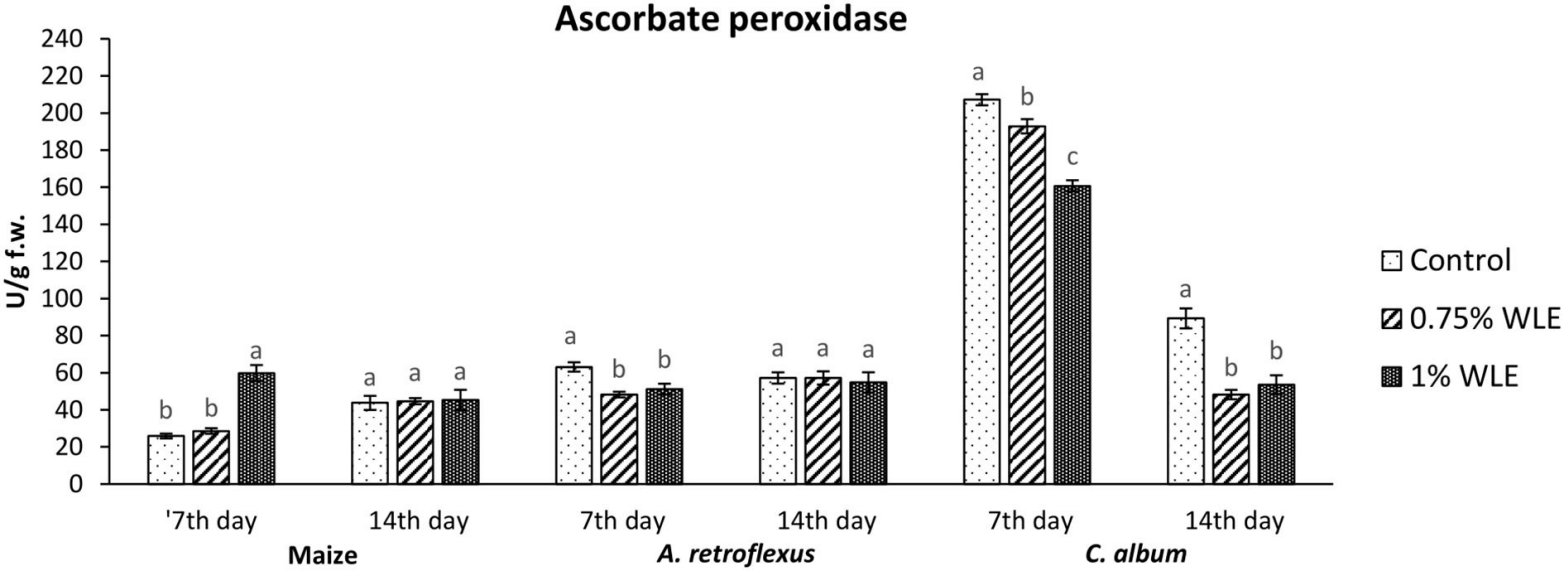
Activity of antioxidant enzyme ascorbate peroxidase (APX) in maize and weeds - *Amaranthus retroflexus* and *Chenopodium album* as a response to treatment with 0.75 and 1% walnut leaf extract, on the 7^th^ and 14^th^ day after treatment. Data are presented as mean ± SD. Values marked with different letters within a group differ significantly based on Tukey's HSD test (*p* ≤ 0.05).

**Figure 6:**
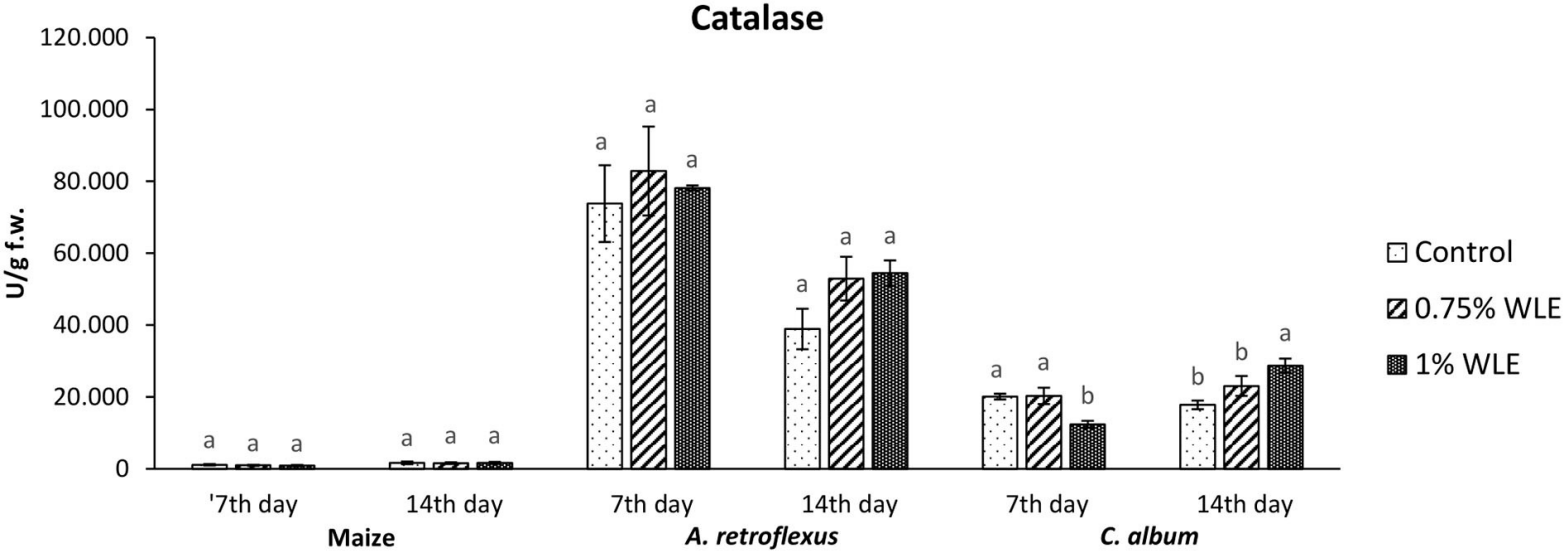
Activity of antioxidant enzyme catalase (CAT) in maize and weeds - *Amaranthus retroflexus* and *Chenopodium album* as a response to treatment with 0.75 and 1% walnut leaf extract, on the 7^th^ and 14^th^ day after treatment. Data are presented as mean ± SD. Values marked with different letters within a group differ significantly based on Tukey's HSD test (*p* ≤ 0.05).

The effects of different concentrations of walnut leaf extract on oxidative stress in the weeds *A. retroflexus* and *C. album*, as well as in maize, are presented in Figures 2–6.

An indicator of oxidative damage to cellular membranes, lipoproteins, and other molecules that contain lipids, caused by increased reactive oxygen species (ROS) contents, is lipid peroxidation (LP). The intensity of LP due to allelopathic stress provoked by treatment with walnut leaf extract is presented in Figure 2. According to Tukey's multiple comparison test, 7 days after treatment (DAT) a statistically significant increase in MDA accumulation was detected after WLE treatments of both tested weeds, as well as maize. Differences between treatments with different walnut leaf extract concentrations were not statistically significant. Higher oxidative damage of lipids occurred in *A. retroflexus* and maize, as the MDA contents in those plants increased by ~30%, while lower lipid damage provoked by WLE treatment was recorded for *C. album*, with an ~10% increase in MDA content. However, 14 days after treatment all tested plants managed to overcome oxidative stress regarding lipid damage as no significant increase in MDA content was recorded on that day.

The enzyme that reduces the content of superoxide radical, usually the first ROS to be formed, undergoes a fast transformation into hydroxyl radical and initiates lipid peroxidation is superoxide dismutase (SOD). Changes in the activity of SOD due to allelopathic stress provoked by treatment with walnut leaf extract are presented in Figure 3. It shows that there were no significant differences in the activity of SOD in maize after treatment with WLE. Regarding *A. retroflexus*, a significant increase in its activity (24%) was recorded on 7 DAT in plants treated with 1% WLE, while on 14 DAT the increase was observed also in plants treated with 0.75% WLE (~10%, similar to both other concentrations). *C. album* was moderately affected by walnut leaf extract, as SOD activity increased 7 DAT (~15%), while there were no significant differences in SOD activity by 14 DAT, compared to control.

Guaiacol peroxidase (GPOD) is an enzyme that uses guaiacol as a substrate to reduce the content of hydrogen peroxide as the main source of hydroxyl radical which initiates lipid peroxidation. Lipid hydroperoxides, from the propagation stage of lipid peroxidation, are also substrates of GPOD. Changes in the activity of GPOD due to allelopathic stress provoked by treatment with walnut leaf extract are presented in Figure 4. The activity of GPOD in maize was overall considerably higher in comparison with its activity in the tested weeds, but there were no significant differences in its activity between control and maize plants treated with WLE. Regarding *A. retroflexus*, a significantly high increase in GPOD activity (82%) was recorded in 7 DAT in plants treated with 1% WLE, but in 14 DAT a significant decrease in this enzyme activity, of ~54%, was observed. In *C. album*, the activity of GPOD was overall very low and it was not affected by treatment with walnut leaf extract.

Ascorbate peroxidase (APX) is an enzyme that uses ascorbic acid as a substrate to reduce the content of hydrogen peroxide as the main source of hydroxyl radical which initiates lipid peroxidation. Changes in the activity of APX due to allelopathic stress triggered by treatment with walnut leaf extract are presented in Figure 5. The activity of APX in maize significantly increased 7 DAT in plants treated with 1% WLE (52%), while 14 DAT there were no significant differences in APX activity, compared to control data. On the contrary, there was a significant decrease in APX activity (~21%) in *A. retroflexus* 7 DAT in plants treated with both concentrations of WLE, while 14 DAT there were no significant differences in APX activity in comparison with control. In *C. album*, the activity of APX also significantly decreased 7 DAT in plants treated with walnut leaf extract (7 and 22%, respectively, after treatment with 0.75% WLE and 1% WLE), and a decrease in APX activity in this weed was even higher 14 DAT (~43% for both concentrations).

Catalase (CAT) is an enzyme that uses hydrogen peroxide, a non-radical ROS, as its substrate. Catalases have a very fast turnover rate, but a much lower affinity for hydrogen peroxide. Changes in the activity of CAT due to allelopathic stress provoked by treatment with walnut leaf extract are presented in Figure 6. The activity of CAT in maize was overall considerably lower than its activity in the tested weeds, but there were no significant differences in the activity between control and maize plants treated with WLE. Regarding *A. retroflexus*, treatment with walnut leaf extract also had no statistically significant effect on CAT activity. In *C. album*, a significant decrease in CAT activity (38%) was recorded 7 DAT in plants treated with 1% WLE, but a significant increase in this enzyme activity, by approximately the same percentage (38%), was observed in those plants 14 DAT.

## Discussion

### *In vitro* evaluation of effects of WLE on seed germination and seedling growth

For preliminary determination of the allelopathic ability of the extracted walnut leaf extract, inhibition of germination and suppression of seedling growth in response to extracting application was initially tested *in vitro* for weeds (*A. retroflexus* and *C. album*). The results of our research indicated that WLE was phytotoxic to both tested weeds. To establish whether the obtained WLE (with proven phytotoxicity to tested weeds) will have a negative impact on a crop in which it would be used for weed control, inhibition of germination and suppression of seedling growth in response to extract application were additionally tested on maize.

Regarding weeds, the allelopathic effect expressed by inhibition of seed germination was more pronounced in *C. album* as its germination rate decreased by more than 50% after treatment with all extract concentrations, i.e., inhibition was ~70% in all bioassays. For *A. retroflexus*, only the highest concentration of walnut leaf extract caused over 50% seed germination inhibition (namely 70%), while inhibition was notably lower after treatment with the lowest and mid concentration of WLE. Inhibition of seed germination by the tested WLE may be associated with phenolic compounds which are allelochemicals that interfere with the germination process by inhibiting amylases and gibberellins. Inhibition of this enzyme and plant hormone then modifies the mobilization of reserves in embryo development (Singh et al., 2009). Besides, as phenolic compounds can interfere with the activity of respiratory enzymes during seed germination (Muscolo et al., 2001), this fact and phenolics-provoked changes in gibberellic acid (Olofsdotter, 2001) are the probable reasons for the germination inhibition noted in weeds. Regarding maize, its seed germination was significantly less affected by walnut leaf extract, as germination inhibition did not exceed 50%, reaching barely ~30% for all three concentrations. This led to a conclusion that, regarding seed germination, maize was less sensitive, i.e., it was generally less affected by the phenolics in WLE considering this parameter. Differences in seed germination among different plant species treated with phenolic compounds were previously reported by De Martino et al. (2012) who found different dose-dependent effects on germination of radish and garden cress, the latter being more sensitive than the former.

Considering seedling growth, walnut leaf extracts significantly reduced the shoot and radical length of both tested weeds similarly. Shoot elongation of both weeds was overall less sensitive to WLE than radical growth. Shoot growth inhibition of *A. retroflexus* was dose-depended, and a significant effect was recorded with higher concentrations, reaching slightly above 50% inhibition in the bioassay testing 1% WLE. Regarding *C. album*, the effect was somewhat lower and was not dose-depended. Shoot elongation inhibition was moderate without statistically significant differences among the three concentrations. Radical growth of both weeds was more affected by WLE treatment, and dose-depended again for *A. retroflexus*, leading to approximately over 60% of growth inhibition. It is interesting that, although maize germination was less sensitive to treatment with walnut leaf extract, phenols in the extract seem to affect seedling growth similar to the tested weeds. Thus, walnut leaf extract had a weaker impact on shoot growth (somewhat under 50% inhibition) of this plant than on radical growth (somewhat above 60% inhibition). The higher overall sensitivity of radical growth is probably because roots are the first plant organ to absorb allelopathic compounds from extracts (Nishida et al., 2005), and root tissue is by its nature more permeable than the rest of the plant (Mominul Islam and Kato-Noguchi, 2013).

Generally, walnut is one of the most recognized allelopathic plants. There are numerous studies on its allelopathic potential that focus on the effect of juglone, as well as other phenolic compounds in its leaf extracts, and all studies reported high inhibitory effects on germination and seedling growth of various cultivated or wild-growing plants (Ercisli et al., 2005; Babula et al., 2014; Medic et al., 2021; Zubay et al., 2021). Phenolic compounds extracted from other plants are also known to influenCe the early seedling growth of various tested plants. Aslani et al. (2014) reported that seed germination, as well as seedling growth of tomato, lettuce, cucumber, rice, and their weeds, were inhibited by a methanolic extract of *Tinospora tuberculata*. The phytotoxic effects of *Peganum harmala* aqueous extracts against early growth of *Avena fetua* and *Convolvulus arvensis* were demonstrated by (Sodaeizadeh et al., 2009).

Regarding individual effects of flavonoids and phenolic acids presented in the tested walnut leaf extract at the highest or mid-dose, (+)-catechin, luteolin, myricetin, rutin, (–)-epicatechin, and genistin, as well as protocatechuic and caffeic acids had a significant contribution to the observed phytotoxicity, as all those phenols have already been proven to have a negative effect on seed germinations and seedling growth of various plants. Golisz et al. (2008) revealed that, although phenolic compounds had a slight effect on the germination of mustard seeds, they inhibited seedling growth, mainly the growth of roots; (–)-epicatechin had a maximum inhibitory effect on the root and rutin had a maximum inhibitory effect on shoot growth, while gallic acid and (+)-catechin also significantly inhibited mustard growth. Krumsri et al. (2020) reported that protocatechuic acid significantly inhibited the growth of cress and barnyard grass seedlings and the effectiveness of this phenol against both plant species was greater in root growth than shoot growth, while Hussain et al. (2020) found that gallic acid, protocatechuic acid, p-hydroxybenzoic acid, p-hydroxybenzaldehyde, vanillic acid, syringic acid, p-coumaric acid, ferulic acid, rutin, luteolin, apigenin, and catechin are all phytochemical compounds from *Acacia melanoxylon* aqueous extract that had significant allelopathic effects.

### *In vivo* evaluation of WLE effects on oxidative stress in growing plants

Allelochemicals applied to soil could be affected by environmental conditions as various abiotic or biotic factors may contribute to their structural modifications, provoking changes in their activities (Jilani et al., 2008). Thus, an evaluation of the allelopathic ability of the obtained walnut leaf extract was carried out in pot experiments. *In vivo* investigation of phytotoxicity of WLE applied at two concentrations was carried out by evaluating oxidative stress in the tested weeds (*A. retroflexus* and *C. album*). Oxidative stress of plants was evaluated 7 days after treatment, and again 14 days after treatment, to see whether the extract with allelopathic potential will have a long-term effect on tested plants or if the plants will overcome the stress induced by allelochemicals from the extracts.

Regarding *A. retroflexus*, the study results showed that treatment with both concentrations of walnut leaf extracts affected lipid peroxidation, which was reflected in an increase in MDA in plants 7 DAT. At that stage, it was obvious that the plant was under stress with overaccumulation of ROS, including superoxide anion (usual the first ROS to be formed) and hydroxyl radical (formed by transformation of superoxide anion and in other ways), and both caused membrane lipid peroxidation. Consequently, the activity of SOD, being the first line of defense against ROS-induced damage, was increased in plants treated with WLE (statistically significant increase at an extract concentration of 1%). Another indicator revealing that redox homeostasis of *A. retroflexus* was affected by treatment with walnut leaf extract was a significant and notably high increase in GPOD activity in plants treated with 1% WLE. The excessive production of ROS is normally accompanied by the activation of enzymatic defenses and GPOD is responsible for reducing hydrogen peroxide as the main source of hydroxyl radical which initiates lipid peroxidation but also uses lipid hydroperoxides, and from the propagation stage of lipid peroxidation as a substrate, it is obvious that this enzyme took the main role in terminating lipid peroxidation in affected plants. Ascorbate peroxidase also has a role in reducing hydrogen peroxide, but the results show that its activity significantly decreased in plants treated with walnut leaf extract at both concentrations, indicating that APX is probably more sensitive to allelochemicals in WLE. As it is known that allelochemicals, besides being directly associated with ROS generation, may also block oxidative enzymes and thus expose plants to high risks of damage (Anwar et al., 2021), it was concluded for *A. retroflexus* that walnut leaf extract caused inactivation of APX, and this decrease in APX activity 7 DAT was compensated with a higher GPOD and SOD activities, indicating that those enzymes can act simultaneously. Catalase activity was not significantly affected by walnut leaf extract in this weed 7 DAT. As this enzyme also reduces hydrogen peroxide but has a much lower affinity, this unchanged activity may be explained by GPOD taking over the main role in hydrogen peroxide scavenging. Fourteen days after treatment with WLE, *A. retroflexus* managed almost to overcome the provoked stress, at least regarding LP. Lipid peroxidation rate at this stage normalized, APX activity was restored, while GPOD activity decreased, indicating that its enhanced activity at the beginning managed to restore the proper balance between hydrogen peroxide (and thus hydroxyl radical) generation and elimination. Only the activity of SOD remained at an elevated level, suggesting that the production of superoxide anions was still elevated, leading to a conclusion that *A. retroflexus* treated with both concentrations of walnut leaf extract was still under oxidative stress, which could most likely cause damage in nucleic acids and proteins or alter carbohydrate metabolism, resulting in cell dysfunction and death.

Regarding *C. album*, the obtained results showed that treatments with both concentrations of walnut leaf extract affected lipid peroxidation in this weed as well. An increase in MDA was recorded at 7 DAT, as WLE caused significant oxidative stress in the plants. The first line of defense against ROS-induced damage was consequently provoked and the activity of SOD increased in plants treated with walnut leaf extract. However, the GPOD activity in this weed was overall very low and was not affected significantly by the WLE treatment. This enzyme is most likely not the leading peroxidase in *C. album* cells. As the activities of APX, as well as CAT, were significantly reduced in *C. album* treated with both concentrations of walnut leaf extract, and yet the level of MDA in plants stabilized after 14 days, it is most likely that in this plant the enzyme glutathione peroxidase, or even natural antioxidants such as vitamin E, took over the inhibition of the propagation step of lipid peroxidation. The obtained LP results show that 14 days after treatment with WLE, *C. album* managed to overcome the provoked stress regarding lipid peroxidation. However, APX activity remained significantly decreased, suggesting that it is possible that allelochemicals from walnut leaf extract blocked or damaged this enzyme. At this stage, the activity of CAT was enhanced, suggesting that the production of hydrogen peroxide was still elevated. This meant that *C. album* treated with WLE leads to a strong enough allelopathy-provoked stress in a way that the scavenging effects of antioxidant enzymes with decreased activity could not prevent the generation of excessive amounts of free radicals that could cause membrane and DNA/protein damage that may eventually result in cell destruction (Cheng and Cheng, 2015).

The phytotoxic effects of plant extracts on recipient plants are mainly a consequence of the activity of allelopathic compounds that act as toxins when applied exogenously, thus inhibiting weed growth (Afzal et al., 2015). Phytotoxins with allelopathic abilities are mostly secondary metabolites such as phenolic acids, flavonoids, quinones, alkaloids, terpenes, and terpenoids (Anwar et al., 2021). Among those bioactive compounds, many families of natural products have been widely studied recently, and they show great potential as future herbicides (Macías et al., 2019). Overall, walnut is a frequently investigated allelopathic plant, but its toxicity is mostly associated with naphthoquinone juglone (Strugstad and Despotovski, 2012). However, juglone, although it is present in considerable amounts in leaves from various walnut cultivars (Cosmulescu et al., 2011), is not the only allelochemical present in *Juglans* species. Oxidative stress in tested weeds, caused by walnut leaf extract, can also be associated with higher contents of phenolics in it, as most of these compounds quantified in WLE have been previously reported as phytotoxic, whereby the overall significant allelopathic potential may be due to their synergistic effect rather than a single constituent.

The most abundant phenolic compound in the obtained walnut leaf extract was flavonoid (+)-catechin which plays a significant role in provoking oxidative stress in *A. retroflexus* and *C. album*, as this compound has been repeatedly proven to possess phytotoxic potential. Inderjit et al. (2008) demonstrated phytotoxic effects of (±)-catechin *in vitro*, in soil, and the field, while Kaushik et al. (2010) revealed that, when applied to the roots of *Arabidopsis thaliana*, it triggers a wave of reactive oxygen species (ROS), leading to a cascade of genome-wide changes in gene expression and, ultimately, death of the root system. Additionally, during the investigation of chemical interactions that occur between three donor species (*Arbutus unedo, Medicago minima*, and *Myrtus communis*) and a receiving species (*Aegilops geniculata*) by studying oxidative stress which occurs in the receiving plant upon treatment with donor extracts it was concluded that catechin negatively affected plant growth when applied at mM concentration (Scognamiglio and Schneider, 2020). The next flavonoid which was present at a high level in our walnut leaf extract was flavone luteolin, which was also reported earlier as a substance with the phytotoxic effect that significantly reduced the frond number and chlorophyll content of *Lemna gibba* plants (Beninger and Hall, 2005). Based on previous studies, it may be assumed that among the flavonoids isolated from walnut leaves in our study, (+)-catechin and luteolin are the main allelochemicals responsible for inducing oxidative stress in tested weeds. Regarding phenolic acids in the obtained walnut leaf extract, protocatechuic acid was the most abundant, and this would not be the first time to report its possible allelopathic ability. Fu et al. (2019) found that a moderate concentration of protocatechuic acid significantly inhibited net photosynthetic rate and stomatal conductance in *Rhododendron delavayi*, while Krumsri et al. (2020) revealed that protocatechuic acid significantly inhibited the growth of cress and barnyard grass seedlings. The next phenolic acid found in significant amounts in our walnut leaf extract was caffeic acid, and it might be the second main allelopathic phenolic acid compound in it. The molecular mechanism of caffeic acid isolated from *Artemisia argyi* and its allelopathic effect on *Setaria viridis* were recently investigated by Chen et al. (2022), who revealed that this phenol significantly induced ROS production, led to MDA accumulation, and disrupted enzyme activities (POD, SOD, CAT) in *S. viridis* leaves. Moreover, their results revealed that caffeic acid inhibited *S. viridis* growth by downregulating multiple genes involved in gibberellin and phytoalexin biosynthesis.

To explore if the walnut leaf extracts had any negative impact on crops on which it would be used for weed control, oxidative stress in maize as a response to treatment with two concentrations of WLE was evaluated 7 and 14 days after treatment. The results showed that maize was only moderately affected by walnut leaf extract treatment. An increase in LP intensity was recorded at 7 DAT, it was coupled with an increase in APX only after 1% of WLE treatment was applied, while the activities of other enzymes remained intact. Further on, 14 days after treatment, the amount of MDA normalized, and the activities of antioxidant enzymes showed no differences between treatment and control, suggesting that the defensive system of maize prevailed. As indicated in numerous earlier studies, the effects of identical extracts are not necessarily the same across various plants as each species has its own response to allelochemicals (Medic et al., 2021).

The results of the present study demonstrated that the obtained walnut leaf extract, which was notably rich in phenolic and flavonoid compounds, had significant negative germination and seedling growth on two tested weeds and also caused significant oxidative stress in weeds grown in pots. This infers that the obtained extract had remarkable allelopathic potential. Given that the extract did not have a significant negative impact on the crop on which it was to be used for weed control, it is possible that the walnut leaf extract obtained in this study could have a promising role in replacing synthetic herbicides.

Not only are the levels of seed germination inhibition and seedling growth suppression usually correlated with the allelopathic extract concentrations (Đordević et al., 2022), but the activation of enzymatic antioxidants in the plants are also strongly dependent on the concentrations of allelochemicals or phytotoxins (Talukder et al., 2020). Lower doses of allelochemicals might provoke the cellular antioxidant system by increasing the activity of enzymes to regain redox homeostasis. However, higher and harmful concentrations of allelochemicals could reduce the activity of enzymatic antioxidants through their inhibition and/or inactivation, resulting in insufficiently scavenged ROS accumulated in cells. In other words, treatment with allelochemicals at low doses (hormesis) will result in the activation of defense mechanisms, while the plant's exposure to higher concentrations of it will most likely result in inhibitory and irreversible modifications in plant growth and metabolism. This should be carefully investigated in further studies that may be required to validate the present results under field conditions.

## Conclusion

The results of the present study demonstrated that the obtained walnut leaf extract with a significant amount of phenolic compounds has remarkable allelopathic potential. This bio extract had a significant negative impact on seed germination and seedling growth of the tested weeds *Amaranthus retroflexus* and *Chenopodium album* and also caused significant oxidative stress in weeds grown in pots. On the other hand, although it appeared that the obtained extract affected maize *in vitro* seedling growth similar to the weeds, maize germination was less sensitive to the WLE treatment, and the extract did not cause significant oxidative stress in the cultivated plant grown in pots. The results demonstrate that walnut leaf extract may have a promising role in replacing synthetic herbicides in maize, a crop in which it could be used for weed control. Additional studies under field conditions and on the ecological role of WLE are required before proceeding toward the development of herbicides based on it.

## Data availability statement

The original contributions presented in the study are included in the article/supplementary material, further inquiries can be directed to the corresponding author.

## Author contributions

TĐ performed the chemical and biochemical experimental procedures and wrote the manuscript. RĐ-P and MS helped in chemical and biochemical experiments, its planning and execution, and reviewed the manuscript. JG-U and MS-K performed bioassays and reviewed the manuscript. LjR and LjŠ helped in bioassay planning and execution and reviewed the manuscript. TĐ and JG-U performed the statistical analysis and data presentation. All authors have read and approved the final manuscript.

## Funding

This investigation was funded by the Ministry of Education, Science and Technological Development of the Republic of Serbia (Grant No. 451-03-9/2022-14/200214).

## Conflict of interest

The authors declare that the research was conducted in the absence of any commercial or financial relationships that could be construed as a potential conflict of interest.

## Publisher's note

All claims expressed in this article are solely those of the authors and do not necessarily represent those of their affiliated organizations, or those of the publisher, the editors and the reviewers. Any product that may be evaluated in this article, or claim that may be made by its manufacturer, is not guaranteed or endorsed by the publisher.
